# Editorial: Systemic Lupus Erythematosus and Antiphospholipid Syndrome

**DOI:** 10.3389/fimmu.2019.00199

**Published:** 2019-02-25

**Authors:** Pier Luigi Meroni, George C. Tsokos

**Affiliations:** ^1^Immunorheumatology Research Laboratory, IRCCS Istituto Auxologico Italiano, Milan, Italy; ^2^Beth Israel Deaconess Medical Center, Harvard Medical School, Boston, MA, United States

**Keywords:** anti-phospholipid yindrome, autoantibodies, T cell, systemic lupus erythematosus, complement

Systemic lupus erythematosus (SLE) and anti-phospholipid syndrome (APS) are frequently discussed together and perceived as two closely related diseases (1). Indeed, up to 40% of SLE patients test positive for phospholipid antibodies (aPL) and a significant proportion of patients with primary APS (i.e., with no associated SLE or other autoimmune diseases) have circulating antinuclear (ANA) and dsDNA/chromatin antibodies (2). Patients with primary APS and SLE share lupus susceptibility genes, yet patients with primary APS do not develop complete SLE even after 10 years of follow-up suggesting a more complex link between the two entities (2–4). Indeed, SLE and APS are distinct entities within the spectrum of systemic autoimmune diseases.

In this collection five manuscripts (Caneparo et al.; Han et al.; Knight et al.; Sakata et al.; Weeding and Sawalha) report pathogenic pathways which appear to operate primarily in patients with SLE. The discussed mechanisms (macrophage differentiation via LXRα, association of DNA methylation with SLE triggering and clinical manifestations, IFN-Inducible Protein 16 as an inflammasome regulator in lupus pathogenesis, endonucleotidase in lupus autoimmunity and vascular damage, up-regulation of TLR7-mediated IFNα production) suggest that multiple heterogeneous pathways operate preferentially in patients with SLE rather than in patients with primary APS. For example, the clinical and histological characteristics of renal involvement in patients with APS definitely differentiate the two entities. In particular, a thrombotic vasculopathy involving medium/large and in some cases small vessels is the main pathogenic mechanism in renal APS in contrast with the inflammatory vasculitis which is characteristic of lupus nephritis (Tektonidou; Turrent-Carriles et al.). Furthermore, involvement of the central nervous system (CNS) is frequent in patients with APS and is mainly linked to vascular thrombotic events while a heterogeneous panel of pathogenic mechanisms contribute to the expression of CNS manifestations in patients with SLE including the presence of NMDR antibodies and the activation of microglia by interferon type I (McGlasson et al.). It is obvious that patients need tailored treatment to address the involved pathogenetic mechanisms.

The fact that several distinct, yet intertwined, pathogenic mechanisms covering every aspect of the immune system operate in patients SLE may explain the multifaceted clinical expression of the disease. It is becoming obvious that SLE comprises diverse diseases each characterized by a dominant operating pathogenetic pathway resulting in unique or shared clinical manifestations (Rekvig). Therefore, the classification of patients along the lines of clinical manifestations cannot serve the patient and definitely has not served the multitude of failed clinical trials (5).

The complexity of the pathogenesis of lupus looms even larger in children with SLE in whom hormonal or extensive environmental factors are not yet major contributors but distinct single gene defects explain the development of SLE. Indeed, as discussed by Lo the list of monogenic SLE patients continues to expand (Lo).

In contrast, the clinical manifestations of patients with APS are easily attributed to thrombophilic events orchestrated by aPL although additional non-thrombotic mechanisms may account for the increased rate of miscarriages (Radic and Pattanaik).

A lot of attention has been paid to aberrant T cell activation pathways in SLE in addition to the tissue damage mediated by immune complex deposition. Several manuscripts in the session of the Journal have actually addressed this issue (Caneparo et al.; Katsuyama et al.; Mizui and Tsokos). SLE “molecular characterization” would be useful for clinicians for a personalized medicine and for better inclusion criteria in clinical trials. In fact, the common biomarkers are not informative enough and we need to enroll more homogenous, along molecular and biochemical lines, populations in the studies and to identify more specific tools for the evaluation of the efficacy of the therapy (5). In contrast, APS is a well-characterized autoantibody-mediated disease but the abnormalities in the cell mediated immune response have been clarified only in part. A manuscript reviewed this issue and discussed the reactivity of T cells against the main antigenic target in APS (i.e., beta2 glycoprotein I), the T-cell epitopes that are recognized and the possible role of T cells in tissue damage (Rauch et al.).

Complement is central in SLE pathogenesis at two levels: luck of the early components C2 and C4 account for the incomplete elimination of autoreactive B cells and lack of C1q for the poor clearance of apoptotic debris whereas excessive activation and generation of the membrane attack complex and the production of C3a and C5a are directly responsible for the execution of tissue damage. APS experimental models support that complement activation takes place in APS as well and it represents a critical step for both aPL-mediated thrombosis and miscarriages. Moreover, there is preliminary evidence for complement activation also in patients. However, the characteristics of complement activation are quite different in SLE and APS further supporting the differences between these two disorders (Tedesco et al.).

We hope that this collection of articles will help readers identify similarities and difference between SLE and APS. More importantly, we hope that they will ask and address critical questions which will advance our understanding of the two entities so that we may serve our patients more effectively.

## Author Contributions

Both PM and GT (i) had a substantial contribution to the conception or design of the work, or the acquisition, analysis or interpretation of data for the work; (ii) drafted the work or revised it critically for important intellectual content; (ii) provided approval for publication of the content, and (iii) agreed to be accountable for all aspects of the work.

### Conflict of Interest Statement

The authors declare that the research was conducted in the absence of any commercial or financial relationships that could be construed as a potential conflict of interest.

## References

[1] Tincani A Andreoli L Chighizola C Meroni PL. The interplay between the antiphospholipid syndrome and systemic lupus erythematosus. Autoimmunity (2009) 42:257–9. 10.1080/08916930902827918 19811269

[2] Andreoli L Pregnolato F Burlingame RW Allegri F Rizzini S Fanelli V. Antinucleosome antibodies in primary antiphospholipid syndrome: a hint at systemic autoimmunity? J Autoimmun. (2008) 30:51–7. 10.1016/j.jaut.2007.11.004 18191541

[3] Yin H Borghi MO Delgado-Vega AM Tincani A Meroni PL Alarcón-Riquelme ME Association of STAT4 and BLK, but not BANK1 or IRF5, with primary antiphospholipid syndrome. Arthritis Rheum. (2009) 60:2468–71. 10.1002/art.24701 19644876

[4] Fredi M Tincani A Yin H Delgado-Vega AM Borghi MO Meroni PL IRF5 is associated with primary antiphospholipid syndrome, but is not a major risk factor. Arthritis Rheum. (2010) 62:1201–2. 10.1002/art.27345 20131273

[5] Wallace DJ. The evolution of drug discovery in systemic lupus erythematosus. Nat Rev Rheumatol. (2015) 11:616. 10.1038/nrrheum.2015.86 26122951

